# Salvage HLA-haploidentical hematopoietic stem cell transplantation with post-transplant cyclophosphamide for graft failure in non-malignant disorders

**DOI:** 10.1038/s41409-021-01323-9

**Published:** 2021-05-09

**Authors:** Michael H. Albert, Mehtap Sirin, Manfred Hoenig, Fabian Hauck, Catharina Schuetz, Rajat Bhattacharyya, Polina Stepensky, Elad Jacoby, Tayfun Güngör, Rita Beier, Ansgar Schulz

**Affiliations:** 1 Department of Pediatrics, Dr. von Hauner Children’s Hospital, University Hospital, LMU Munich, Munich, Germany; 2 Department of Pediatrics and Adolescent Medicine, University Medical Center Ulm, Ulm, Germany; 3 German Centre for Infection Research (DZIF), Munich, Germany; 4 Department of Pediatrics, Medizinische Fakultät Carl Gustav Carus, Technische Universität Dresden, Dresden, Germany; 5 Haematology Oncology Service, Department of Paediatric subspecialties, KK Women’s and Children’s Hospital, Bukit Timah, Singapore; 6 Faculty of Medicine, Hebrew University of Jerusalem, Jerusalem, Israel; 7 The Department of Bone Marrow Transplantation, Hadassah Medical Center, Jerusalem, Israel; 8 Division of Pediatric Hematology Oncology and BMT, The Edmond and Lily Safra Children’s Hospital, Sheba Medical Center, and Sackler Faculty of Medicine, Tel Aviv University, Tel Aviv, Israel; 9 Department of Hematology/Oncology/Immunology, Gene-therapy, and Stem Cell Transplantation, University Children’s Hospital Zürich – Eleonore Foundation & Children’s Research Center (CRC), Zürich, Switzerland; 10 Department of Pediatric Hematology and Oncology, University Duisburg-Essen, Essen, Germany; 11 Department of Pediatric Hematology and Oncology, Hannover Medical School, Hannover, Germany

**Keywords:** Biostatistics, Translational research

## Abstract

Graft failure requires urgent salvage HSCT, but there is no universally accepted approach for this situation. We investigated T-cell replete haploidentical HSCT with post-transplantation cyclophosphamide following serotherapy-based, radiation-free, reduced intensity conditioning in children with non-malignant disorders who had rejected their primary graft. Twelve patients with primary or secondary graft failure received T-cell replete bone marrow grafts from haploidentical donors and post-transplantation cyclophosphamide. The recommended conditioning regimen comprised rituximab 375 mg/m^2^, alemtuzumab 0.4 mg/kg, fludarabine 150 mg/m^2^, treosulfan 20–24 g/m^2^ and cyclophosphamide 29 mg/kg. After a median follow-up of 26 months (7–95), eleven of twelve patients (92%) are alive and well with complete donor chimerism in ten. Neutrophil and platelet engraftment were observed in all patients after a median of 18 days (15–61) and 39 days (15–191), respectively. Acute GVHD grade I was observed in 1/12 patients (8%) and mild chronic GVHD in 1/12 patients (8%). Viral reactivations and disease were frequent complications at 75% and 42%, respectively, but no death from infectious causes occurred. In summary, this retrospective analysis demonstrates that a post-transplantation cyclophosphamide-based HLA-haploidentical salvage HSCT after irradiation-free conditioning results in excellent engraftment and overall survival in children with non-malignant diseases.

## Introduction

Graft failure (GF) is a rare but severe complication after hematopoietic stem cell transplantation (HSCT). GF is relatively infrequent in leukemia patients and after matched related donor HSCT, but occurs in up to 10% of patients with non-malignant disorders after cord blood, matched unrelated (MUD) or haploidentical HSCT [1–5].

Primary GF is defined as failure of sustained hematopoiesis (absolute neutrophil count <500/µl, hemoglobin <80 g/L, platelets <20 G/L) by day +28 or +42 (cord blood) after HSCT. Secondary GF occurs after initial donor cell engraftment, usually later than d + 28. Graft rejection is defined as immune-mediated rejection of donor cells and can result in primary as well as secondary GF [6]. Poor graft function on the other hand describes cytopenia occurring after d + 28 in the presence of donor chimerism, which can often be successfully treated with a stem cell boost [7].

The etiology of GF is multifactorial, with contributing factors such as underlying disease, alloimmunization with anti-HLA antibodies, intensity of conditioning, drug toxicity, viral infections, infused hematopoietic stem cell dose, graft manipulation, serotherapy, and degree of HLA-(mis)matching [5, 8, 9]. Patients with non-malignant diseases have not been exposed to chemotherapy prior to conditioning for their first HSCT and are therefore at higher risk for GF than patients with malignant diseases [1, 4, 5]. As a graft-versus-leukemia effect is not needed to cure these diseases, in vivo and ex vivo T-cell depletion of the graft is often more stringent in these HSCTs and may contribute to graft rejection/failure, especially in patients with inborn errors and intact/residual NK and T cell function.

Prolonged pancytopenia and immunosuppression after GF represent a life-threatening emergency. If available, an unconditioned CD34+-selected stem cell boost from the original donor may be successful in select patients with poor graft function and predominant donor chimerism [10]. In situations of acute GF/rejection and in the absence of a cryopreserved autograft, a salvage second allogeneic HSCT is usually the only lifesaving treatment. However, there is no universally accepted approach for donor choice or conditioning regimen, and recruitment of a second unrelated donor is often not possible in a timely manner. At least one HLA-haploidentical family donor is usually readily available for almost all patients, especially for pediatric patients [11]. Recently, it was demonstrated that the outcomes and GVHD rates of haploidentical HSCT with post-transplant cyclophosphamide (pTCy) were comparable to those with matched related or unrelated donors in adult and pediatric patients with leukemia [12, 13], as well as in pediatric patients with inborn errors [14–16]. PTCy is potently tolerogenic by selectively depleting alloreactive T cells responsible for GVHD and graft rejection, while sparing stem cells, regulatory T cells and non-alloreactive resting T cells, which are needed for adaptive immunity e.g. against viruses [17]. Two groups have recently reported encouraging results in children with leukemia and GF using a low-dose total body irradiation (TBI) or total-lymphoid irradiation (TLI) based re-conditioning strategy [18, 19]. However, the appropriateness of ionizing irradiation in this situation can be questioned for several reasons including particular concerns about long-term toxicity especially in young children with non-malignant disease [20].

We therefore hypothesized that the combination of a chemotherapy-based, immunoablative re-conditioning containing serotherapy followed by T cell replete haploidentical bone marrow with pTCy would be able to overcome engraftment barriers in children with non-malignant disorders and GF. We designed a salvage HSCT protocol combining these properties, and present here the results of an international retrospective analysis of this approach in seven centers.

## Methods

### Definitions

Engraftment was defined as the first of three consecutive days to reach an absolute neutrophil count (ANC) of >500/µl or platelets of >20.000/µl without transfusion support. Primary GF was defined as not having achieved neutrophil engraftment until day +28. Secondary GF was defined as loss of donor engraftment after day +28, with or without associated ANC < 500/µl. Patients with anti-HLA antibodies directed against an HLA antigen present in the donor were defined as anti-HLA donor-specific antibody (DSA) positive. Staging of acute GVHD was performed according to modified Glucksberg criteria and chronic GVHD was staged according to NIH consensus standards [21, 22].

### Treatment recommendations

**Fig. 1:**
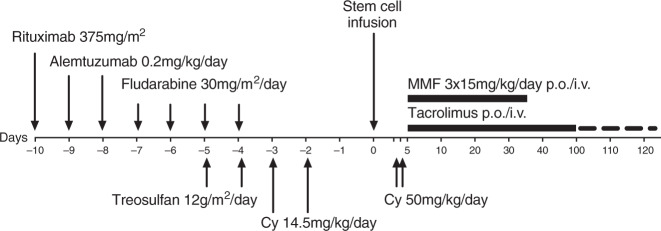
Recommended conditioning regimen. Cy: cyclophosphamide; MMF: mycophenolate mofetil.

The treatment protocol was designed for patients with non-malignant diseases and GF after a previous allogeneic HSCT. Recommended conditioning consisted of rituximab 375 mg/m^2^ (day -10), alemtuzumab 0.4 mg/kg (days −9 to −8), fludarabine 150 mg/m^2^ (days −7 to −3), treosulfan 24 g/m^2^ (days −5 to −4) and cyclophosphamide 29 mg/kg (days −3 to −2), followed by unmanipulated bone marrow from HLA-haploidentical family donors (Fig. 1). GVHD prophylaxis was recommended as cyclophosphamide 2x50mg/kg on days +3 and +4, tacrolimus from day +5, tapered after day +100, plus mycophenolate mofetil (MMF) from day +5 to day +35. Erythrocyte or plasma depletion was performed for AB0 incompatibility. Supportive care was carried out according to local institutional standards and contained antiviral, mold-active antifungal and *Pneumocystis jirovecii* prophylaxis in all centers.

### Data collection

The treatment recommendations for haploidentical salvage HSCT were devised in Munich, Germany, and disseminated upon request to the participating centers. We retrospectively collected data from 12 consecutive GF patients transplanted between 1st of January 2012 and 31st of July 2020 at six centers (Munich, Ulm, Essen, Singapore, Jerusalem, and Ramat Gan), representing all pediatric patients with non-malignant diseases presenting with GF at these centers during that time period. Patient caregivers consented to retrospective file-based data collection via the ethically approved “Pädiatrisches Register für Stammzelltransplantation” (PRST) in Munich, Ulm, and Essen, and local ethics approval was obtained for anonymized data transfer at the other centers if required by local standards.

## Results

### Patient characteristics, previous HSCTs and graft failure

We report on 12 patients from seven centers, who underwent salvage haploidentical HSCT following the proposed regimen. All patients suffered from non-malignant diseases, either inborn errors of immunity (*n* = 7) or bone marrow failure syndromes (*n* = 5). All had undergone previous HSCT at a median age of 4.5 years (range 1.0–14.7) from a matched unrelated (MUD; *n* = 4) or a haploidentical mismatched family donor (MMFD; *n* = 8) after reduced intensity (RIC; *n* = 6) or myeloablative (MAC; *n* = 6) conditioning regimens. None of the patients had detectable donor-specific anti-HLA-antibodies against their first donor. All patient and previous HSCT characteristics are detailed in Table 1.

**Table 1:** Details of previous HSCT and characteristics of graft failure.

pt	Diagnosis	Age at first HSCT (years)	Sex	Donor	Conditioning	Stem cell source	Graft manipulation	Type of graft failure (GF)	Chimerism at time of GF (source)	Second HSCT/ boost from same donor	Conditioning	Graft	Graft manipulation	Engraftment
1	IL10 receptor α deficiency	5.5	f	MMFD (mother)	FLU 160, MEL 70, TT 10, ATG-F 60	PB	CD3/CD19 depletion	primary	0% (PB)	Yes	FLU 120, TT 5, CY 60, TBI 2, ATG-G 4.5	PB	CD34 selection	no
2	MDS-RCC	5.1	f	MMFD (mother)	FLU 160, MEL 70, TT 10, ATG-F 30	PB	CD3/CD19 depletion	primary	0% (PB)	No	–	–	–	–
3	CGD	11.8	m	MUD	BU_AUC_ 55.000, FLU 160, CAMP 0,9	BM	–	secondary	0.5% (PB)	No	–	–	–	–
4	SCN	1.2	m	MUD	BU_AUC_ 80.531, FLU 160, CAMP 0,6	BM	–	primary	0% (PB)	No	–	–	–	–
5	SCN	1.2	m	MUD	BU_AUC_ 84.350, FLU 160, CAMP 0,6	BM	–	secondary	0% (PB)	Yes	none	BM	none	no
6	MDS-RCC	11.7	m	MUD	FLU 160, TT15, ATG-F 45	BM	–	primary	0% (PB)	No	–	–	–	–
7	MDS-RCC	5.2	m	MMFD (father)	FLU 160, MEL 140, TT10, ATG-F 30	PB	TCRα/β, CD19 depletion	primary	0% (PB)	No	–	–	–	–
8	HIGM	1.1	m	MMFD (mother)	BU_AUC_ 80.600, FLU 160, TT 10, ATG-G 10	PB	CD34 selection	primary	40% (PB)	No	–	–	–	–
9	HLH-Perforin	1.0	m	MMFD (father)	TREO 36, FLU 150, TT 10, ATG-F 15, RTX 200	PB	TCRα/β, CD19 depletion	secondary	12% (PB)	No	–	–	–	–
10	CAMT	3.0	m	MMFD (father)	BU 16, FLU 180, TT 10, ATG-G 10	BM	- (pTCy)	secondary	0% (BM/PB)	No	–	–	–	–
11	SAA	3.8	f	MMFD (mother)	TBI 2, FLU 160, CY 120, TT 10, ATG-G 7,5	PB	TCRα/β, CD19 depletion	primary	0% (PB)	Yes	TNI 7, FLU 120, TT 5, CY 60, ATG-G 4,5	PB	TCRα/β, CD19 depletion	no
12	CGD	14.7	m	MMFD (father)	TREO 42, FLU 150, TT 10, CAMP 0,5	PB	CD3/CD19 depletion	secondary	<5% (PB)	No	–	–	–	–

*ATG-F* rabbit anti-T-cell-lymphoglobuline (Grafalon®), *ATG-G* rabbit anti-T-cell-lymphoglobuline (Thymoglobuline®), *BM* bone marrow, *BU* busulfan (mg/kg), *BUAUC* busulfan with area-under-the-curve pharmacokinetic measurement (ng*h/ml), *CAMP* alemtuzumab, *CAMT* congenital amegakaryocytic thrombocytopenia, *CD* cluster of differentiation, *CGD* chronic granulomatous disease, *CY* cyclophosphamide (mg/kg), *FLU* fludarabine (mg/m^2^), *HIGM* Hyper-IgM syndrome, *HLH* hemophagocytic lymphohistiocytosis, *MDS-RCC* myelodysplastic syndrome-refractory cytopenia of childhood, *MEL* melphalan, *PB* peripheral blood, *MMFD* mismatched family donor, *MUD* matched unrelated donor, *RTX* rituximab (mg/m^2^), *SAA* severe aplastic anemia, *SCN* severe congenital neutropenia, *TCR* T-cell receptor, *TT* thiotepa (mg/kg).

Seven patients presented with primary and five with secondary GF. After GF, two patients had received a second conditioned HSCT from the original donor; one received an unconditioned bone marrow boost with previously cryopreserved cells. Neither resulted in detectable donor engraftment. Three patients had a diagnosis of GF before d + 28 (pts 6, 7, and 11). All of them experienced a short-lived increase in white blood cells with some degree of donor chimerism followed by high fevers and a drop of counts and disappearance of donor chimerism. Therefore, the diagnosis of GF was “prematurely” established before d + 28 in these patients. All patients had <50% (*n* = 2) or undetectable (*n* = 10) peripheral blood donor chimerism at time of GF (Table 1).

### Salvage HSCT

**Fig. 2:**
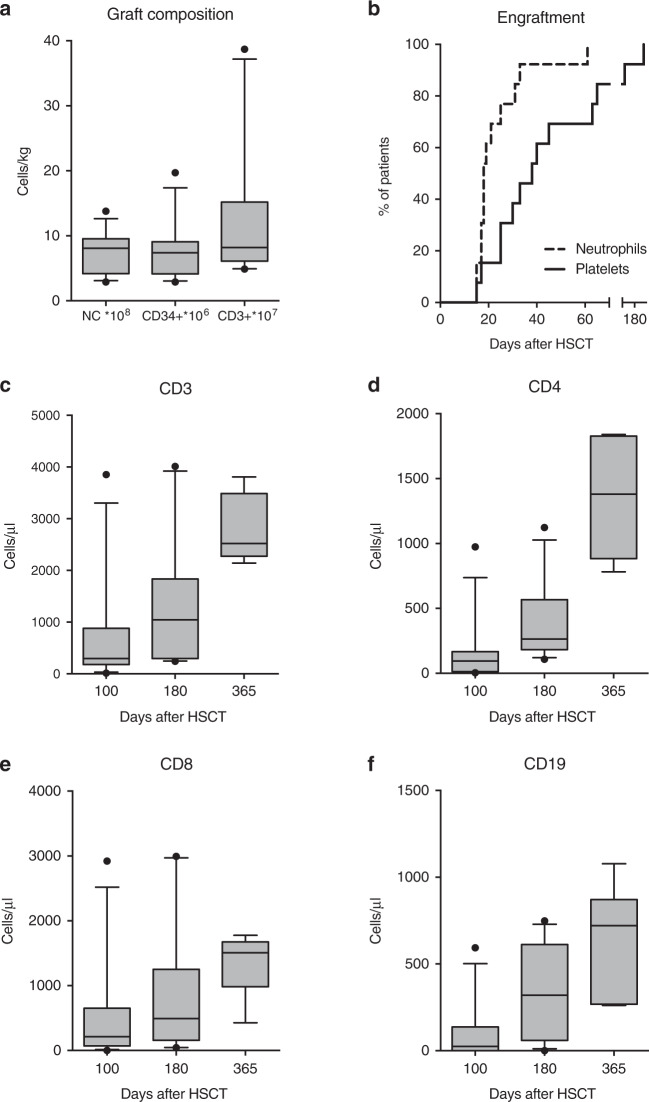
Graft composition, engraftment and cellular immune reconstitution. Graft composition (**A**), engraftment kinetics (**B**), and immune reconstitution (**C**–**F**) after salvage HSCT.

At a median of 46 days (29–115) after their previous HSCT, 12 patients underwent salvage HSCT with an unmanipulated graft from an HLA-haploidentical family donor (*n* = 11) or a mismatched unrelated donor (8/10 HLA match; *n* = 1). An alternative donor was chosen for the salvage HSCT in 11/12 patients. Slight modifications of the suggested regimen (Fig. 1) were possible by institutional preference, but a backbone of a treosulfan/fludarabine-based, radiation-free, reduced intensity conditioning followed by T-cell replete mismatched donor grafts and GVHD prophylaxis with pTCy, calcineurin inhibitor and MMF was maintained throughout the cohort. Patient 11 received ATG instead of alemtuzumab because the latter was not available in his country. Conditioning consisted of fludarabine, reduced-dose treosulfan, low-dose cyclophosphamide and T cell-directed serotherapy in all patients. Eleven patients also received a single dose of rituximab. Detailed dosing of the conditioning regimen can be found in the methods section and Table 2. The grafts were unmanipulated bone marrow (*n* = 11) or PBSC (*n* = 1) grafts with a median total nucleated cell count of 9.2 × 10^8^/kg (2.9–13.8), CD34^+^ count of 7.8 ×10^6^/kg (2.9–19.7) and CD3^+^ count of 8.4 × 10^6^/kg (4.9–38.7) (Fig. 2). GVHD prophylaxis consisted of cyclophosphamide 50 mg/kg/d on days +3 and +4, followed by mycophenolate mofetil (MMF) and tacrolimus (*n* = 11) or cyclosporine A (CSA, *n* = 1) starting on day +5. Immunosuppression was stopped after a median time of 110 days after salvage HSCT (range 56–252).

**Table 2:** Salvage HSCT characteristics.

pt	age at salvage HSCT (years)	time from previous HSCT (days)	donor	HLA match	DSA	conditioning	graft source	TNC (×10^8^/kg)	CD34 (×10^6^/kg)	CD3 (×10^6^/kg)	GVHD prophylaxis
1	5.7	43	MMFD (father)	5/10	ND	TREO 24, FLU 150, CAMP 0.6, CY 29, RTX 375	BM	9.9	8.2	7.9	CY, TAC, MMF
2	5.2	38	MMFD (father)	5/10	ND	TREO 24, FLU 150, CAMP 0.4, CY 29, RTX 375	BM	8.9	5.1	8.2	CY, TAC, MMF
3	12.2	115	MMFD (father)	6/10	No	TREO 36, FLU 150, CAMP 0.6, CY 29, RTX 375	BM	7.3	7.8	4.9	CY, TAC, MMF
4	1.3	44	MMFD (father)	6/10	No	TREO 24, FLU 150, CAMP 0.4, CY 29, RTX 375	BM	13.8	19.7	15.2	CY, TAC, MMF
5	1.4	74	MMFD (father)	6/10	Yes	TREO 24, FLU 150, CAMP 0.4, CY 29, RTX 375	BM	9.6	6.8	31.1	CY, TAC, MMF
6	11.8	35	MMFD (mother)	5/10	ND	TREO 24, FLU 150, CAMP 0.4, CY 29, RTX 375	BM	2.9	2.9	6.1	CY, TAC, MMF
7	5.3	30	MMFD (mother)	5/10	ND	TREO 24, FLU 150, CAMP 0.4, CY 29, RTX 375	BM	3.9	7.4	5.1	CY, TAC, MMF
8	1.2	51	MMFD (brother)	7/10	ND	TREO 20, FLU 160, CAMP 0.6, CY 29, RTX 375	BM	5.8	13.9	12.7	CY, TAC, MMF
9	1.2	48	MMFD (father)	6/10	No	TREO 20, FLU 150, CAMP 1.0, CY 29	PB	9.4	10.0	38.7	CY, TAC, MMF
10	3.0	49	MMFD (mother)	5/10	No	TREO 36, FLU 150, CAMP 0.6, CY 29, RTX 375	BM	9.4	8.0	8.1	CY, CSA, MMF
11	4.0	29	MMFD (father)	5/10	No	TREO 24, FLU 120, ATG-G 5, CY 29, RTX 375	BM	5.1	3.8	8.5	CY, TAC, MMF
12	14.8	47	MMUD	8/10	Yes	TREO 24, FLU 150, CAMP 0.4, CY 29, RTX 375	BM	3.6	4.5	ND	CY, TAC, MMF

*CSA* cyclosporine A, *DSA* donor-specific anti-HLA-antibodies, *MMF* mycophenolate mofetil, *MMUD* mismatched unrelated donor, *ND* not done.

### Engraftment, survival, and immune reconstitution

Neutrophil and platelet engraftment after salvage HSCT were observed in all patients after a median of 18 days (15–61) and 39 days (15–191), respectively (Fig. 2). After a median follow-up of 26 months (7–95) eleven of twelve patients (92%) are alive, well and disease-free with complete donor chimerism in 10/11. One patient died on day +259 after salvage HSCT from radiologically determined chronic lymphocytic inflammation with pontine perivascular enhancement responsive to steroids (CLIPPERS) syndrome [23] (Table 3). Cellular immune reconstitution was timely in our cohort with a median of 298/µl CD3^+^ (12–3852), 89/µl CD4^+^ (4–974), 281/µl CD8^+^ (2–2922) T cells and 30/µl CD19^+^ (0–593) B cells on day +100, 1050/µl CD3^+^ (271–4010), 276/µl CD4^+^ (108–1123), 582/µl CD8^+^ (43-2994) T cells and 328/µl CD19^+^ (0–748) B cells on day +180, and 2635/µl CD3^+^ (2142–3806), 1622/µl CD4^+^ (782–1840), 1592/µl CD8^+^ (428–1776) T cells and 800/µl CD19^+^ (261–1078) cells on day +365 (Fig. 2). All patients were off immunoglobulin substitution at last follow-up and no patient required substitution after d + 180.

**Table 3:** Outcome of salvage HSCT.

pt	Alive	Whole blood donor chimerism at last f/u	Follow-up (months)	Acute GVHD	Chronic GVHD	Viral reactivation	Viral disease	Other complications	Cause of death	Cessation of immunosuppression (d+)
1	Yes	100	95	–	–	CMV, ADV	BK hemorrhagic cystitis	–	n/a	56
2	Yes	100	63	–	–	–	–	Engraftment syndrome (ARDS), bacteremia (Streptococcus mitis)	n/a	72
3	Yes	100	50	–	–	–	–	–	n/a	110
4	Yes	100	53	–	–	–	ADV (enteritis)	–	n/a	110
5	Yes	100	6	–	–	CMV, ADV	ADV (systemic)	Aspergillosis	n/a	74
6	Yes	100	6	–	–	ADV, HHV6	–	Sepsis, toxic nephropathy	n/a	124
7	Yes	100	8	–	–	CMV	–	Sepsis, colitis (Clostridium difficle)	n/a	124
8	Yes	100	10	I (skin)	mild (skin)	CMV, HHV6	Norovirus (enteritis)	Sepsis, deep skin infection (Pseudomonas),	n/a	252
9	Yes	30–40	30	–	–	CMV	–	Sepsis (Escherichia coli), bacteremia (coagulase-neg. Staphylococcus)	n/a	228
10	Yes	100	21	–	–	CMV, ADV	–	–	n/a	92
11	Yes	100	37	–	–	CMV, ADV, BK, HHV6	BK hemorrhagic cystitis	Sepsis (Enterococcus faecium), fungal skin infection (Trichosporon asahi)	n/a	100
12	No	100	9	–	–	CMV, ADV	–	Idiopathic pneumonitis (d + 226) treated with etanercept	Death (d + 259) due to CLIPPERS syndrome with intracranial bleeding and consecutive hydrocephalus	240

*ADV* adenovirus, *ARDS* acute respiratory distress syndrome, *CLIPPERS* chronic lymphocytic inflammation with pontine perivascular enhancement responsive to steroids, *CMV* cytomegalovirus, *HHV6* Human herpesvirus 6.

### Toxicity and GVHD

Toxicity greater than CTCAE grade 2 was observed in one patient with elevated transaminases ≥3 times above the upper limit of normal. No patient developed veno-occlusive disease (VOD; *n* = 2 with defibrotide prophylaxis) or thrombotic microangiopathy. Viral reactivation with asymptomatic viremia was the most frequent complication and occurred in nine out of twelve patients (75%) with detection of CMV (*n* = 8), ADV (6), HHV6 (3), and BK virus (2). Five patients (42%) had symptomatic viral infections (ADV 2, BK 2, norovirus 1). All viral reactivations/infections either resolved spontaneously or were successfully treated. Bacterial sepsis or bacteremia were observed in five and two patients, respectively. Other complications are detailed in Table 3. GVHD was almost negligible with acute GVHD grade I (skin stage 2) observed in 1/12 patients (8%) and mild chronic GVHD (skin only) in 1/12 patients (8%), which was resolved at last follow-up in all patients (Table 3).

## Discussion

GF after allogenic HSCT is associated with high mortality rates because of the risk for severe infections during prolonged aplasia and organ dysfunction after conditioning ^1-3^. The risk for GF is reported to be up to 3-fold higher in non-malignant diseases. GF without autologous reconstitution of hematopoiesis is a life-threatening emergency requiring a timely salvage HSCT. In this report, we describe 12 patients with non-malignant diseases, who received a salvage HSCT with T replete grafts from mismatched donors using a modified Baltimore protocol without total body irradiation [24]. All patients engrafted and 11/12 (92%) survived and are alive and well.

In the event of GF, a salvage transplantation is crucial to establish a functioning donor hematopoiesis as quickly as possible. In most instances, it is challenging to find a matched alternative donor in a timely manner. Unrelated umbilical cord blood grafts are usually readily available but implicate a high risk for second GF and infection-related mortality because of prolonged immune reconstitution [25]. Therefore, HLA-haploidentical related donors represent an attractive alternative donor source. Because immune-mediated processes are known to play a role in the rejection of donor stem cells by the recipient, we aimed at maximizing the tolerogenic capacity of the conditioning regimen, while reducing the toxicities associated with intensive myeloablation, which is not needed in patients with near aplastic marrow. In addition to the immunosuppressive quality of fludarabine, we incorporated serotherapy directed at remaining immunocompetent recipient T-, B- and NK-cells. The dose of alemtuzumab in our regimen was low and applied rather early before graft infusion in order to minimize the effect on donor graft facilitating cells. While the low absolute dose administered not later than day -8 can be expected to result in sub-T-cell-lympholytic levels at the time of graft infusion [26, 27], it cannot be excluded that because of the low lymphocyte counts of the recipients a longer than usual half-life of alemtuzumab and rituximab could have resulted. In theory, this could explain the relatively high rate of viral reactivations observed in this cohort, but there was no apparent detrimental effect on quantitative T- and B-cell reconstitution and blood levels of serotherapy agents were not monitored in our patients. Alemtuzumab not only targets T cells but also B cells and plasma cells [28]. Still, we chose to recommend the additional application of rituximab—also in DSA negative patients—due to the anticipated but hypothetical need for maximal disruption of humoral anti-donor immunity of the recipient including the hypothetical role of non-HLA anti-donor antibodies [29]. However, the non-controlled nature of the study does not allow drawing a firm conclusion on whether the addition of serotherapy was relevant for the excellent overall outcome in these patients.

A number of case series of successful salvage HSCT for GF employing different conditioning regimens and donor sources have been published, but there is no universally accepted approach for this situation. Various small series reported encouraging results with overall survival (OS) ranging from 36% to 63%, in adults with hematologic malignancies [30–32] to 96% in adults with aplastic anemia and matched sibling donors (MSD) [33]. Of particular interest, Teltschik et al. reported on eleven pediatric patients predominantly with leukemia who experienced GF [18]. The salvage HSCT was performed after total nodal irradiation (TNI)-based conditioning using T-cell depleted peripheral blood stem cell grafts from HLA-haploidentical donors. All patients engrafted and the OS was 68% with little severe acute (11% grade III), no severe chronic GVHD, and transplant related mortality (TRM) of 11% [18]. Park et al used a similar approach in ten pediatric patients with primary GF after HSCT for severe aplastic anemia, leukemia, or MDS. They also infused T-cell depleted grafts (with CD3 or CD3/CD19 depletion) from MMFD after fludarabine/cyclophosphamide conditioning—with the addition of TBI in three patients—and reported a 2-year OS of 88% with ≥ grade III acute GVHD of 25%, no severe chronic GVHD, and a TRM of 10% [34]. These results compare well with our cohort, which exhibits an OS of 92%, no severe acute or chronic GVHD, and a TRM of 8%. Of note, all three approaches resulted in 100% engraftment after salvage HSCT, but in our cohort, this was achieved without irradiation as part of the conditioning regimen. Because of the perceived long-term detrimental effects, irradiation remains controversial in children, especially in those with non-malignant diseases. Furthermore, in the setting of bone marrow aplasia after GF, it might be worth considering that irradiation implicates that the patient has to leave the protective isolation of the transplant unit several times, and that smaller infants need sedation or general anesthesia to tolerate the irradiation procedure. The very low rates of severe acute and chronic GVHD using haploidentical donors in all three cohorts is encouraging, given the degree of HLA-disparity. Of note, in our cohort the only patient who died had a mismatched unrelated donor. This donor was chosen because the mother was a disease carrier, the father was the donor for the previous HSCT, and a donor change was intended. It remains speculative whether an HLA-haploidentical donor with one genoidentical haplotype would have been advantageous in this setting.

Like any other retrospective, multi-center study, our analysis is prone to selection bias and heterogeneity of patients and transplant centers. However, all pediatric patients with non-malignant diseases presenting with GF at the participating centers during this period were included into this analysis, limiting a particular selection bias in this respect.

It is well established that donor T cells play an important role in promoting engraftment [35], which is why we chose a T-cell replete instead of an in vivo T-cell depleted graft. In fact, two of our patients had undergone a second conditioned in vivo T-cell depleted MMFD HSCT, with either one of the regimens proposed by Teltschik et al and Park et al. Both did not result in engraftment. The role of changing the donor for a second HSCT remains controversial [36]. In our cohort, all but one patient received their salvage HSCT from a different donor. This was intended in order to account for hypothetical, undetectable donor-specific immunity of the recipient. Even though the only patient who was re-transplanted with the same donor is also the only one with incomplete donor chimerism at last follow-up, it remains speculative whether any donor-specific immunity outside of DSA may have played a role here.

Immune reconstitution after HSCT with in vitro T-cell depletion with CD3/CD19 depletion or CD34 positive selection is generally expected to be slow [37]. Accordingly, Park et al observed high rates of viral reactivation and disease in 90% and 50% of patients, respectively [34]. T cell receptor alpha/beta depleted PBSC grafts may result in faster T-cell recovery [38]. However, exact rates of viral reactivations/infections per patient were not reported by Teltschik et al, but the rate of infectious toxicity of grade 3–4 CTCAE was 48% [18]. In the pTCy protocol, a T-cell replete graft is transfused, alloreactive T cells are depleted in vivo by cyclophosphamide, and resting memory T cells responsible for infection control are presumably spared [17]. Nevertheless, viral reactivations were also frequent in 75% of patients in our cohort, and 42% experienced viral disease either successfully treated with antivirals or spontaneously resolving without sequela or chronic morbidity. This may be explained by the overall immunosuppressive nature of this regimen and/or by some degree of post HSCT immunosuppression caused by the serotherapy in our regimen as discussed above. However, quantitative immune reconstitution of T cells in our cohort was comparable to what is expected after a MSD or MUD HSCT and faster than after in vitro T cell depletion by CD34 positive selection [38, 39]. Likely, because of the low dose of treosulfan and the absence of irradiation, there was little acute toxicity apart from infections, especially no VOD nor severe mucositis. This stands in contrast to the TNI-based protocol employed by Teltschik et al where grade 3-4 mucositis was observed in 84% of patients [18].

In summary, we demonstrate that a pTCy-based HLA-haploidentical HSCT after an irradiation-free conditioning results in excellent engraftment and overall survival in this cohort of children with non-malignant diseases, who had experienced GF. Even though this is a non-comparative and retrospective analysis with a limited number of patients, it shows that early salvage HSCT from MMFD with pTCy is a feasible approach in the event of GF.
